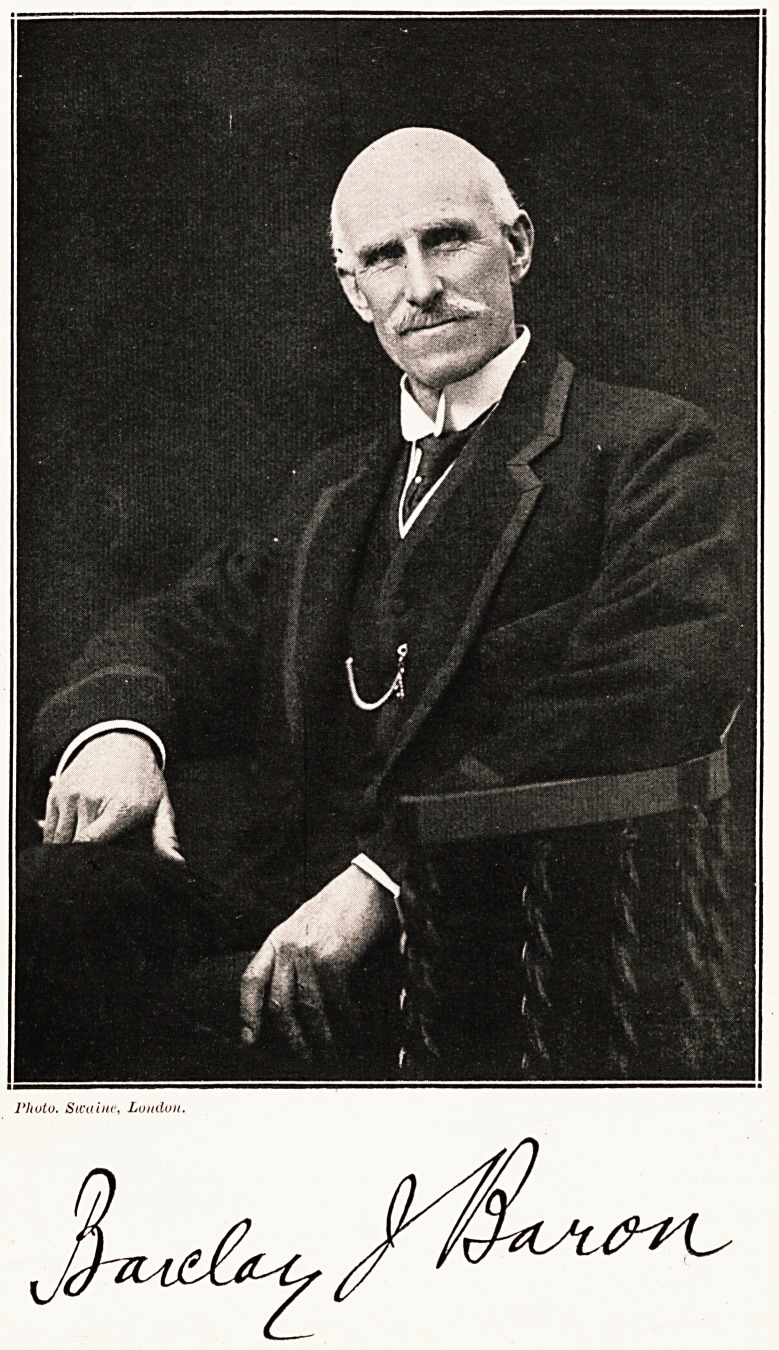# Barclay Josiah Baron, Knight

**Published:** 1919

**Authors:** 


### Obituary.

### BARCLAY JOSIAH BARON, KNIGHT.

THE loss to Bristol from the death of Sir Barclay Baron is indeed a very heavy one, both as the loss of a wise municipal councillor and as a member of our own profession. As the great civic loss which Bristol has sustained by his death has been referred to at length in the daily press, we do not intend to refer to it here, except to say that his mental ability and power as Lord Mayor was only such as those who knew him in his professional life might have anticipated. We would rather in this notice refer to his very brilliant intellectual qualities as manifest in our knowledge of him as a colleague in the work of the medical profession.

That Sir Barclay Baron's intellectual attainments were of the highest order was first shown by the success of his university career at Edinburgh, where he was the first student of his year out of several hundred, and took the Ettles Scholarship in 1881, and several prizes and medals ; and later by the character of his work in his own special branch of medicine, and his ability as an exponent of it, and by his grasp of, and judgment on, matters requiring important consideration, He combined with this high intellectual attainment, that gift of fluent speech which made him such an interesting companion in private, and such an able public speaker, and those social qualities which made it a pleasure to so many of his colleagues to have intercourse with him. We well remember the mntual reciprocation of constant friendly feeling between Sir Barclay and two of his colleagues who have also been lost to us—Dr. Markham Skerritt and Dr. Aust Lawrence. They served to form a very effective combination for mutual help, good comradeship and influence.

Sir Barclay Baron was born in 1857, and was 61 at the time of his death. He was a member of an old Quaker family of Plymouth. For eighteen years he had charge of the Throat and Nose Department at the General Hospital, and of this department he was the founder. He did a very large amount of most valuable work for the hospital in this department, and he continued this work until the demands of a large private practice in this branch of medicine made it impossible. His advice in difficult cases, in his special line of work, was very widely sought by the medical profession in Bristol and elsewhere in the West of England, and during the period of the war he was appointed as one of the consulting larnygologists for the Southern Command. At Edinburgh he was Assistant to the Professor of Pathology, and at one time he lectured on Pathology in the Bristol Medical School, and fortunate were the students who had so fluent a lecturer.

Old members of the Bristol Medico-Chirurgical Society will remember the time when Sir Barclay Baron was its genial President, and his very interesting Presidential Address, and workers in his own special department who were members of the British Laryngological Association will recall his Presidency of that Society also. He was also President of the Bath and Bristol Branch of the British Medical Association.

But Sir Barclay Baron had other interests besides municipal and profession work. He was an active agent in the establishment of the Playgoers' Club, where various dramatic works were read and discussed. He had for many years been in close connection with many actors, as their adviser with regard to voice production and those throat and larynx troubles to which they are liable. He was also much interested in art, and was a member of the Savage Club, a local society, mainly of artists. His ability as a public speaker made him a very welcome guest at the dinners of some of those societies founded by natives of various districts, such as the Caledonian and the Devonian Societies.

It may interest readers of this Journal to know something of our colleague's last illness. He was pulling down the dead branch of a tree in his garden on May 25th, when it broke off before he expected, and he fell, striking the lower part of the left side of his chest on a large stone. He was much distressed after the fall, but not more than a fracture of some ribs would account for, and he obtained considerable relief when the injured side of the chest was supported by strapping, and there was no emphysema or other evidence of damage to the lung, or abdominal injury, but he retched several times. The day following the accident his temperature rose to 102°, and he suffered from marked headache, and some abdominal distension. The pyrexia continued for some days, the temperature keeping at 100° to 101°, and was not explainable in connection with the accident. It was suggested to Sir Barclay that if he had not had the accident this pyrexial condition would be labelled influenza, as almost all mysterious pyrexial conditions now are, but a few days later he expressed his belief that what had been suggested in joke was really true, and he traced the infection to riding in a crowded omnibus with many sick-looking, coughing people three days before the accident, and this he said was his usual incubation period for influenza, of which he had many attacks. After the subsidence of this pyrexial condition he was most unfortunately attacked in the middle of the night with a horrible condition of severe pain in the lumbo-sacral region so tha the literally could not move an inch in the bed. This was an apyrexial condition, and was greatly relieved by aspirin. It lasted almost until his death, for it was only an hour before he died that he was able to sit up in bed for the first time for several days. He had had many of these attacks of lumbago. For this condition, and the anorexia due to his inability to have any action of the bowels on account of the impossibility of the slightest movement in bed, he was seen by a physician on Friday evening, June 6th. He examined his abdomen and found nothing abnormal, and a perfectly normal pulse, and did not entertain any idea that he was in a critical condition. At 1.0 a.m. next morning Sir Barclay woke up and complained of headache. This was relieved by a cup of tea. At 3.0 a.m. he sat up in bed, which he had not been able to do for several days, and as soon as he raised himself into the sitting position he felt faint, and his breathing became laboured, and by 4.0 a.m. he was dead.

## Figures and Tables

**Figure f1:**